# Notch Signaling Pathway Is Activated by Sulfate Reducing Bacteria

**DOI:** 10.3389/fcimb.2021.695299

**Published:** 2021-07-15

**Authors:** Sudha B. Singh, Cristina N. Coffman, Amanda Carroll-Portillo, Matthew G. Varga, Henry C. Lin

**Affiliations:** ^1^ Biomedical Research Institute of New Mexico, New Mexico VA Health Care System, Albuquerque, NM, United States; ^2^ Division of Gastroenterology and Hepatology, Department of Medicine, University of New Mexico, Albuquerque, NM, United States; ^3^ Medicine Service, New Mexico VA Health Care System, Albuquerque, NM, United States

**Keywords:** *Desulfovibrio vulgaris* (*DSV*), sulfate reducing bacteria (SRB), Notch1, pro-IL-1β, NICD, DAPT, SOCS3

## Abstract

Sulfate Reducing Bacteria (SRB), usually rare residents of the gut, are often found in increased numbers (called a SRB bloom) in inflammatory conditions such as Inflammatory Bowel Disease (IBD), pouchitis, and periodontitis. However, the underlying mechanisms of this association remain largely unknown. Notch signaling, a conserved cell-cell communication pathway, is usually involved in tissue development and differentiation. Dysregulated Notch signaling is observed in inflammatory conditions such as IBD. Lipolysaccharide and pathogens also activate Notch pathway in macrophages. In this study, we tested whether Desulfovibrio, the most dominant SRB genus in the gut, may activate Notch signaling. RAW 264.7 macrophages were infected with *Desulfovibrio vulgaris* (DSV) and analyzed for the expression of Notch signaling pathway-related proteins. We found that DSV induced protein expression of Notch1 receptor, Notch intracellular domain (NICD) and p21, a downstream Notch target, in a dose-and time-dependent manner. DSV also induced the expression of pro-IL1β, a precursor of IL-1β, and SOCS3, a regulator of cytokine signaling. The gamma secretase inhibitor DAPT or Notch siRNA dampened DSV-induced Notch-related protein expression as well the expression of pro-IL1β and SOCS3. Induction of Notch-related proteins by DSV was not affected by TLR4 -IN -C34(C34), a TLR4 receptor antagonist. Additionally, cell-free supernatant of DSV-infected macrophages induced NICD expression in uninfected macrophages. DSV also activated Notch pathway in the human epithelial cell line HCT116 and in mouse small intestine. Thus, our study uncovers a novel mechanism by which SRB interact with host cells by activating Notch signaling pathway. Our study lays a framework for examining whether the Notch pathway induced by SRB contributes to inflammation in conditions associated with SRB bloom and whether it can be targeted as a therapeutic approach to treat these conditions.

## Introduction

Sulfate reducing bacteria (SRB) are anaerobic, gram negative, commensal residents of the colon in humans and other animals (Gibson et al., 1988). The dominant genus among the SRB family in the intestine is *Desulfovibrio* (Loubinoux et al., 2002). SRB has been mainly enumerated in the feces in various studies (Gibson et al., 1991; Gibson et al., 2006). The localization of SRB in the gut remains largely unexplored. A study showed that *Desulfovibrio* was mainly localized in the right colon in healthy subjects (Nava et al., 2012). In another study, *Desulfovibrio* species were also shown associated with colonic mucin in healthy subjects and in ulcerative colits patients (Earley et al., 2015). A bloom of SRB is found in inflammatory conditions such as inflammatory bowel disease (IBD), colitis, pouchitis and periodontitis (Singh and Lin, 2015). It has been suggested that high concentration of SRB in the intestine may be correlated to the inflammation and could be a possible indicator of occurrence of IBD **(** Kushkevych et al., 2019). In addition, we have previously shown an increase in the density of *Desulfovibrio* in small intestine in a longitudinal study in mice treated with a cocktail of antibiotics (Singh et al., 2017). However, the mechanisms of how SRB interact with host cells remain largely unexplored. Better understanding of interaction of SRB with host cells is critical to unraveling the relationship between SRB bloom and inflammatory diseases and to identify novel targets for treating these conditions.

Notch signaling is a highly conserved cell-cell communication pathway known to be important in development and tissue differentiation (Artavanis-Tsakonas et al., 1999). In the intestine, Notch signaling has been shown to participate in determination of cell fate (Noah and Shroyer, 2013). Canonical Notch signaling is activated upon binding of Notch ligands expressed on signal-sending cells to Notch receptors expressed on the signal-receiving cells (Bray, 2016). This is followed by a two-step cleavage of the Notch receptor. The first cleavage occurs at the extracellular domain of Notch receptor and is catalyzed by α ADAM (A Disintegrin and Metalloprotease) metalloprotease converting enzyme (S2 cleavage). A second cleavage step occurs intracellularly in the signal-receiving cell by gamma secretase protease (S3 cleavage) resulting in the release of Notch intracellular domain (NICD). NICD then translocates to the nucleus and binds to CBF1–Suppressor of Hairless–LAG1 (CSL or RBP-J), which acts as a transcription repressor. Upon binding of NICD to CSL, the co-repressors are displaced and co-activators such as mastermind protein (MAML1) are recruited to the complex. This leads to transcriptional activation of Notch target genes such as *hes, hey, p21*, and *myc* (Borggrefe and Oswald, 2009; Kopan and Ilagan, 2009). Four Notch receptors (Notch1-4) and five Notch ligands (DLL1, DLL3, DLL4, Jagged1 (Jag1), and Jagged 2 (Jag2) have been identified (D’Souza et al., 2010).

Notch signaling is activated in macrophages by LPS (Tsao et al., 2011), *Mycobacterium bovis BCG* (Narayana and Balaji, 2008), and *Ehrlichia chaffeensis* (Lina et al., 2016). Notch signaling promotes an inflammatory cascade in macrophages (Fung et al., 2007; Levi, 2017), and blocking Notch attenuates proinflammatory cytokine IL-1β production in LPS-stimulated cells (Tsao et al., 2011). In this study, we tested the hypothesis that SRB may induce Notch signaling in macrophages. We treated RAW 264.7 murine macrophages with DSV and analyzed Notch signaling markers. We also analyzed the expression of pro-IL-1β, a precursor of mature IL-1β and SOCS3 (Suppressor Of Cytokine Signaling 3), a regulator of cytokine signaling, in response to DSV in the absence or presence of Notch inhibition. We confirmed our findings in primary bone marrow-derived macrophages, epithelial cell lines, and in mouse intestine. We also tested whether Notch activation in DSV-infected macrophages could be transmitted to uninfected cells by soluble factors in a paracrine manner.

## Materials and Methods

### Animals

Five-week-old female C57BL/6 mice were purchased from Charles River Laboratory (Wilmington, DE). Upon arrival, animals were housed in polypropylene cages and placed on a 12-hour light/dark cycle and kept on a standard rodent diet (Harlan Teklad Laboratory Diets). Mice were subjected to one-week acclimatization period. Procedures were approved by the Institutional Animal Care and Use Committee at the New Mexico VA Health Care System following guidelines provided by the Guide for the Care and Use of Animals of the National Research Council.

### *Desulfovibrio vulgaris* (DSV) growth

*Desulfovibrio vulgaris* Hildenborough (ATCC 29579, Manassas, VA) was grown anaerobically in Hungate tubes using LeGall’s media. Media composition: 37.39 mM NH_4_Cl, 28.16 mM Na_2_SO_4_, 16.61 mM MgSO_4_, 2.87 mM K_2_HPO_4_, 0.4% of Sodium Lactate (60% syrup), 0.2% of FeSO_4,_ and 0.4% yeast extract. Cultures were grown for ~72 hours in 5 ml aliquots at 37°C. Bacteria were counted using Quantom Tx cell counter (Logos Biosystems, South Korea) and also with a Petroff Hausser counting chamber (Hausser Scientific). Before infection, bacteria were pelleted and resuspended in phosphate buffered saline solution. Heat killed bacteria were generated by autoclaving DSV. Volume equivalent of MOI20 in live bacteria was used for infecting RAW cells.

### DSV Gavage

DSV was orally gavaged in mice at the density of 10^9^ bacteria/100µl in PBS. Control group was gavaged with PBS alone (N=6/group). One hour post gavage, animals were euthanized and 1/3^rd^ regions of small intestinal tissues corresponding to proximal duodenum, mid jejunum, and distal ileum were collected in Trizol (Thermofisher Scientific). Intestines were flushed once with PBS to remove luminal content before collecting the tissues.

### Cell Culture and Treatments

RAW 264.7 murine macrophages and HCT116 human colonic epithelial cells were purchased from ATCC (Manassas, VA). RAW cells were grown in DMEM+10% FBS. HCT116 cells were grown in McCoy’s 5a Medium Modified+10% FBS. No antibiotics were added to the culture media. Cells were grown at 37°C in a humidified incubator with 5% CO_2_. Prior to the day of infection, 8x10^5^ cells were plated in a 6-well plate. Cells were treated with DSV at various MOI and times. For DAPT treatment (D5942, Sigma Aldrich), cells were pretreated overnight with various DAPT concentrations followed by infection with DSV. In control cells, equivalent volume of DMSO was added as a vehicle control for DAPT. DMSO itself was found to have no effect on the expression of Notch proteins when compared to no DMSO control (data not shown).

### Bone Marrow-Derived Macrophage (BMM) Isolation and Culture

Bone marrow-derived macrophages (BMM) were isolated from female C57/Bl6 mice (Charles River). Following euthanasia, femurs were extracted and cleaned of all tissue. The marrow was extracted in a sterile environment by clipping the epiphyses of both ends of the femur, and flushing the marrow with a 27G needle and 3 mL of BMM growth media (RPMI1640 (Invitrogen), 10% Fetal Bovine Serum (ATCC), 20% L929 supernatant (gifted by Dr. Eliseo Castillo, UNM), 5% Pen-Strep (Invitrogen). Marrow was pipetted to break up clumps and then passed through a filter to remove all large material. Cells were then centrifuged at 1000 *x g* for 5 min. The pellet was resuspended in fresh BMM media, cells were counted, and seeded into cell culture flasks at 1 x 10^6^/ml. Cells were used between 7 and 10 days. The day prior to experiments, BMMs were trypsinized, counted (8x10^5^ cells), and reseeded onto 6 well plates with antibiotic free media.

### siRNA Transfection

Three Notch siRNAs (mouse specific silencer select) were purchased from Thermofisher. Cells were transfected with 35nM combined siRNAs by electroporation using nucleofector kit V (Lonza), using manufacturer’s instruction. Cells were plated in 6-well plates for 24 hours. The following day, cells were scraped, counted (8x10^5^), and re-plated for 24 hours (total time of transfection 48hrs). Cells were infected with DSV at MOI 20 for 7 hours and harvested for Western blotting.

### Western Blot

Cells were lysed in Lysis buffer (ThermoFisher: 87787) containing protease and phosphatase inhibitors (ThermoFisher: 1861281) for 20 mins on ice. Tissue samples in Trizol were processed as per manufacturer’s instructions (Thermofisher Scientific). Protein concentration was determined with a Bradford reagent (Bio-Rad). Fifty µg of protein samples from cells and 10µg of proteins from tissues were run on SDS-PAGE (4-20% tris-glycine) and transferred to nitrocellulose membranes. Membranes were blocked in 5% milk in PBS-T (0.1%Tween 20) for 30 mins followed by overnight incubation in antibodies against Actin (Cell Signaling: 4970), Notch1 (abcam 52627), NICD (Cell Signaling: 4147), p21 (Cell Signaling: 64016), SOCS3 (Cell Signaling: 52113), and IL-1β (Cell Signaling: 12426). Antibodies were diluted as recommended by the manufacturer. Blots were incubated with secondary antibodies (Cell Signaling: 7074) at room temperature for 1 hour (dilution of 1:2000) and developed using enhanced Chemiluminiscence HRP signal (ThermoFisher: 32106, 34577, 37075).

### ELISA

Mice proximal small intestinal tissues from control or DSV-treated animals were analyzed for the presence of IL-1β using a mouse ELISA IL-1β kit as per manufacturer’s protocol (Sigma RAB0274). Values represent average IL-1βpg/mg of protein (N=6/group). Data is plotted as Mean ± SEM.

### Paracrine Effect of DSV-Induced Notch Signaling

DSV was cultured in Legall’s medium. Bacteria were pelleted and resuspended in PBS and then added to the macrophages at MOI 20. RAW cells were then infected with either DSV or PBS for 7 hours. A third condition was also included in the experiment which consisted of only DSV and the culture medium (DMEM+10% FBS) and no RAW cells. Following infection, supernatants from 1) PBS control cells (Control sup), 2) DSV infected cells (DSV+RAW cells Sup), and 3) DSV alone in culture medium (DSV-RAW cells sup) were collected and passed through a 0.2 µm filter and added overnight to a fresh plate of uninfected cells. The following day, recipient cells were harvested and analyzed by western blotting for NICD expression. In another set, the recipient cells were pre-treated with various concentrations of DAPT overnight. Medium was removed and replaced with filtered 1) control sup, 2) DSV+RAW cell sup or 3) DSV-RAW cell sup. Fresh DAPT was again added to the recipient cells along with the culture supernatants and cells were incubated overnight (~18hrs). The following day, cells were harvested for Western blotting.

### Quantitative PCR

RNA was prepared using RNeasy Mini Kit (Qiagen: 74106F). The cDNA was prepared using Applied Biosystems High Capacity cDNA Reverse Transcription Kit (ThermoFisher: 4368814). Real-time quantitative PCR was performed with the Applied Biosystems QuantStudio 7 Flex (ThermoFisher) using the Applied Biosystems TaqMan Universal PCR Master Mix (ThermoFisher: 4304437). Notch receptor and ligand gene expression for Notch 1 (Mm00435249_m1), Notch 2 (Mm00803077_m1), Notch 3 (Mm1345646_m1), Notch 4 (Mm00440525_m1), DLL 1 (Mm01279269), DLL 3 (Mm00432854), DLL 4 (Mm00444619_m1), Jag 1 (Mm00496902_m1), Jag 2 (Mm01325629_m1), and IL-1β (Mm00434228_m1) were obtained using Applied Biosystems TaqMan probes (ThermoFisher). qPCR conditions as defined by Thermofisher were as follows:

**Table:** 

Thermal cycling conditions		
Stage	Temp (°C)	Time (mm:ss)
Hold	50	2:00
Hold	95	10:00
Cycle (40 Cycles)	95	00:15
	60	1:00

Host gene expression was analyzed by calculating fold change values using 2-^ddct^ method. Gene expression of 18S was used as a housekeeping control (Applied Biosystems, ThermoFisher).

### Statistical Analysis

All graphs were generated using Graph Pad Prism 5. Data is plotted as Mean ± SEM relative to control. Each experiment was conducted at least three independent times. For western blot analysis, within each independent experiment, three biological replicates were used and combined to represent one treatment group. For data analysis, we compared differences between the control and DSV treated groups using a two-tailed t-test. One-way ANOVA with a post-hoc Dunnett’s multiple comparison test was used for comparing the difference between three or more groups. P values <0.05 were considered significant.

## Results

### Expression of Notch1 Signaling Proteins, SOCS3, and pro-IL-1β in Response to DSV

**Figure 1:**
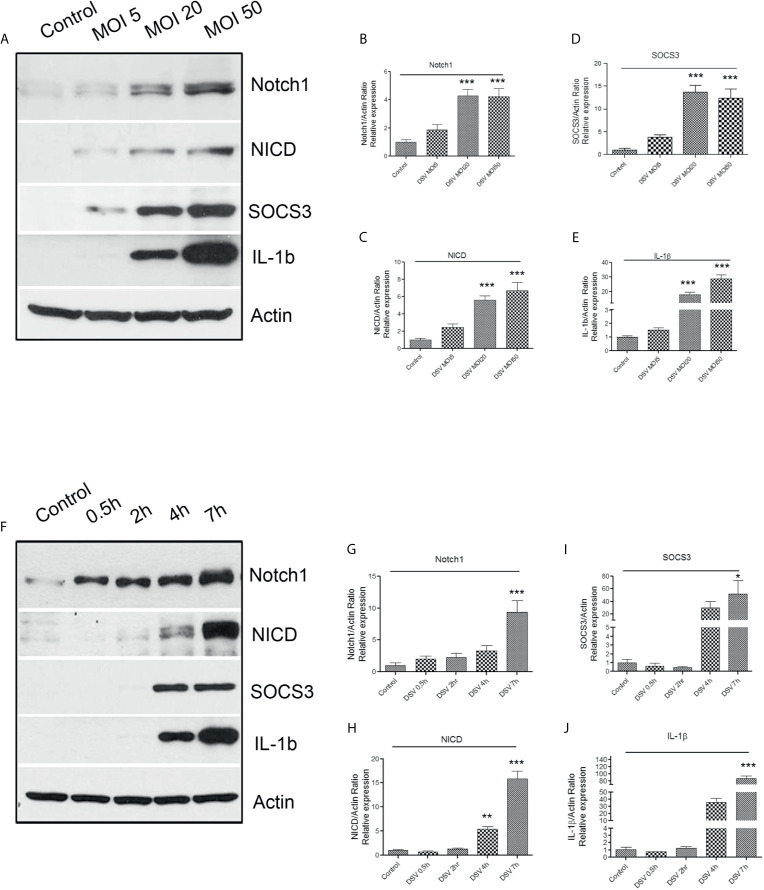
DSV induces dose and time-dependent activation of Notch1 signaling markers, SOCS3, and pro-IL-1β expression **(A)** RAW cells (8x10^5^) were infected with DSV at MOI 5, 20 or 50 for 7 hours. Cells were lysed and protein lysate was prepared. Fifty μg of protein lysate was separated on SDS-PAGE and analyzed for Notch1, NICD, SOCS3, and IL-1β by Western blotting. Actin was used as a loading control. **(B–E)** Quantification of Western blots. Blots were quantified with ImageJ by analyzing the ratio of protein of interest/Actin. Data represents Mean ± SEM from at least three independent experiments. Values were normalized to control. **(F)** Cells were infected with DSV (MOI 20) for 0.5, 2, 4, and 7 hours. Fifty μg of protein lysate was separated on SDS-PAGE and analyzed for Notch1, NICD, SOCS3, and IL-1β by Western blotting. Actin was used as a loading control. **(G–J)** Quantification of Western blots. Blots were quantified with ImageJ by analyzing the ratio of protein of interest/Actin. Values were normalized to control. Data represents Mean ± SEM from at least three independent experiments. One-way ANOVA was used to determine the statistical significance. Values were compared to control, with a post-hoc Dunnett’s test. *p < 0.05, **p < 0.01, ***p < 0.001.

First, we tested whether DSV activated Notch1 signaling pathway in RAW 264.7 macrophages. Cells were treated with DSV for 7 h using multiplicity of infection (MOI) of 5, 20, or 50. Protein expression was analyzed by Western blotting. The 7-hour time point was selected based on the reported induced expression of Notch1 receptor in macrophages by LPS (Tsao et al., 2011). We found that DSV induced Notch1 expression in a dose- dependent manner (**Figures 1A, B**) with a significant induction observed at MOI 20 (4.27 ± 0.46.08, p<0.001) and at MOI 50 (4.22 ± 0.55, p<0.001), when compared to untreated control cells (1.00±0.17). Similarly, DSV significantly induced the expression of Notch intracellular domain (NICD) at MOI 20 (5.58 ± 0.51, p<0.001) and MOI 50 (6.69 ± 0.93, p<0.001) (**Figure 1C**). Suppressor of cytokine signaling 3 (SOCS3) has been shown to be activated downstream of Notch (Narayana and Balaji, 2008) and is involved in the regulation of cytokine signaling. We found that SOCS3 expression was significantly increased in cells treated with DSV compared to control cells at MOI 20 (13.67 ± 1.49, p<0.001) (**Figure 1D**) and at MOI 50 (12.33±2.022, p<0.001). We also tested whether DSV induced the expression of pro-IL-1β (~37kDa) cytokine in macrophages. We observed a dose-dependent induction of pro-IL-1β with significant increase at MOI 20 (18.08 ± 1.59, p<0.001) and at MOI 50 (28.81 ± 2.81, p<0.001) when compared to control cells (1.00±0.09) (**Figures 1A, E**). In subsequent experiments, we used MOI 20 as the lowest dose that induced a significant activation of Notch pathway and pro-IL-1β expression.

Next, we analyzed activation of Notch signaling in response to DSV at various time points (**Figure 1F**). We found that DSV induced Notch1 expression as early as 0.5 h with maximum expression at 7 h (9.398 ± 1.82, p<0.001) when compared to control (1.00±0.39) (**Figure 1G**). Expression of NICD induced by DSV was significant at 4 h (5.38 ± 0.52, p<0.01) which further increased at 7 h (15.86 ± 1.55, p<0.001) (**Figure 1H**). Expression of SOCS3 (**Figure 1I**) and pro-IL-1β (**Figure 1J**) was also induced by DSV in a time-dependent manner with maximum expression observed at 7 h (SOCS3: 52.04 ± 21.40, p<0.05; IL-1β:86.63 ± 7.17, p<0.001).

In addition to live DSV, we found that heat killed DSV (autoclaved) also induced NICD, p21, and pro-IL-1β protein expression at MOI20, 7 hr post infection compared to control (p<0.05) (**Figure S1**).

### mRNA Expression of Notch Ligands and Receptors in Response to DSV

**Figure 2:**
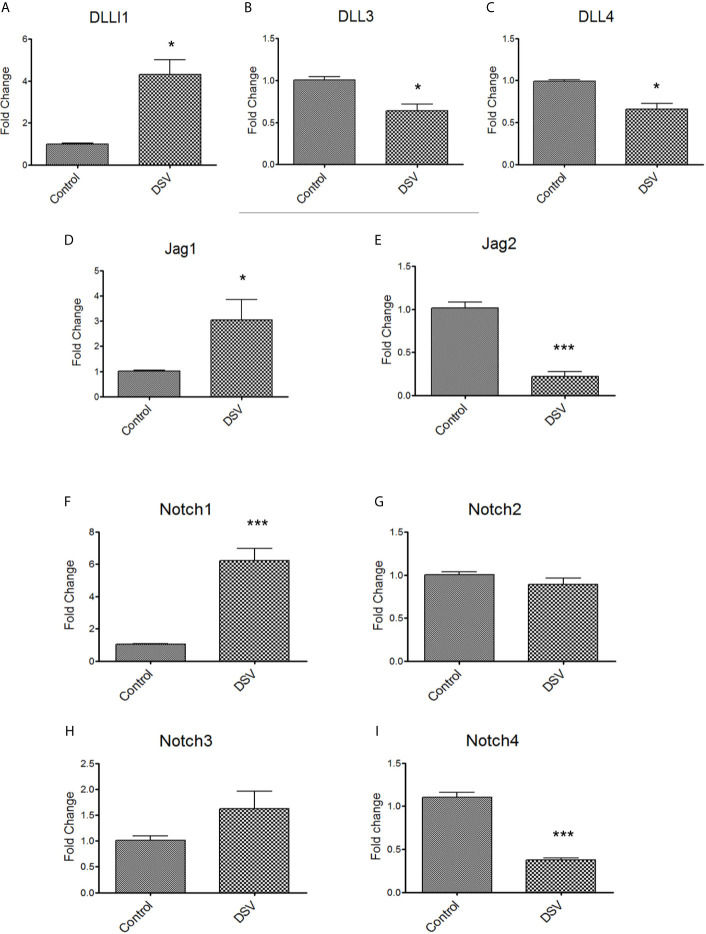
DSV differentially induces gene expression of Notch ligands and receptors RAW cells were treated with DSV **(**MOI 20) for 7hrs and harvested, RNA was isolated and cDNA synthesized. QPCR was carried out to determine the gene expression of **(A)** DLL1, **(B)** DLL3, **(C)** DLL4, **(D)** Jag1, **(E)** Jag2, **(F)** Notch1, **(G)**, Notch2, **(H)** Notch3, and **(I)** Notch4 using Taqman probes for respective genes. Relative fold change expression in DSV treated cells compared to untreated control cells was calculated using 2^-ddct^ method using 18s gene expression as the housekeeping control. A two-tailed t-test was used to determine the statistical difference between control and DSV-treated cells. *p < 0.05, ***p < 0.001.

We carried out quantitative PCR (qPCR) to analyze the gene expression of Notch ligands (DLL1, DLL3, DLL4, Jag1, Jag2) and receptors (Notch1-4) in cells infected with DSV (**Figure 2**). We found that DSV caused a significant increase in DLL1 (DSV: 4.30 ± 0.70 *vs* control: 1.00 ± 0.04, p<0.05) (**Figure 2A**). In contrast, a mild but significant reduction in the expression of DLL3 (DSV: 0.63 ± 0.07 *vs* control: 1.00 ± 0.04, p<0.01) (**Figure 2B**) and DLL4 (DSV: 0.65 ± 0.06 *vs* control 0.98 ± 0.02, p<0.05) (**Figure 2C**) was observed in DSV treated cells. An increase in Jag1 (DSV: 3.04 ± 0.80 *vs* control: 1.00 ± 0.03, p<0.05) (**Figure 2D**) but a decrease in Jag2 (DSV: 0.22 ± 0.053 *vs* control: 1.01 ± 0.06, p<0.001) (**Figure 2E**) were observed in DSV treated cells in comparison to the control cells. We also analyzed the expression of Notch receptors in response to DSV. As expected from our western blot data (**Figure 1G**), we observed a significant induction of Notch1 expression by DSV (DSV: 6.21 ± 0.75 *vs* control: 1.05 ± 0.02, p<0.001) (**Figure 2F**). However, there was no effect on the gene expression of Notch2 or Notch3 by DSV (compared to control cells) (**Figures 2G, H**). In contrast, DSV inhibited Notch4 expression (DSV: 0.37 ± 0.025 *vs* control: 1.10 ± 0.05, p<0.001) (**Figure 2I**). These results suggest that DSV differentially affects the gene expression of Notch ligands and receptors, causing induction of some while inhibiting the expression of others.

### DSV-Induced Notch Signaling, SOCS3, and IL-1β Expression Is Inhibited by Gamma Secretase Inhibitor DAPT and by siRNA Against Notch1

**Figure 3:**
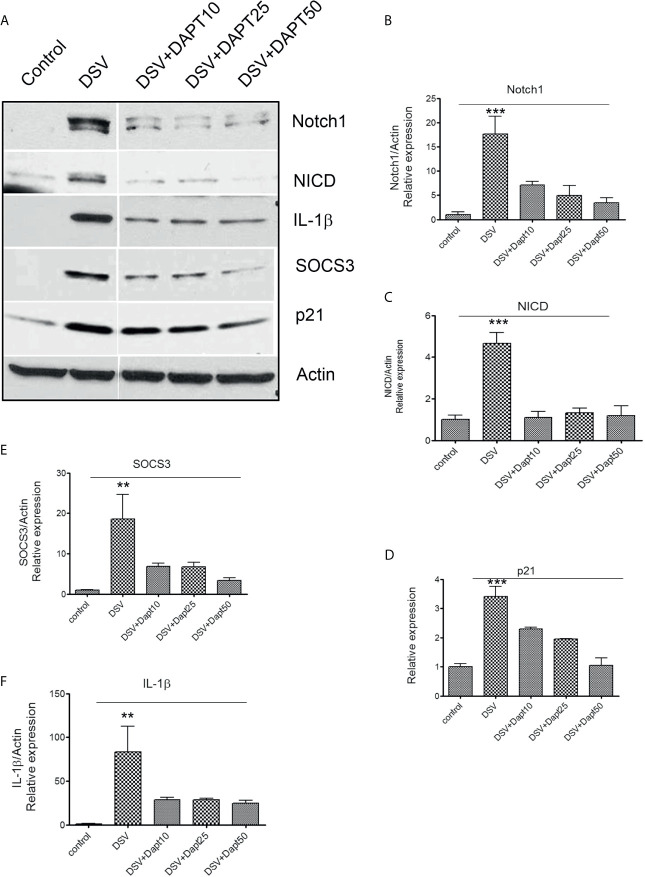
DSV-induced Notch signaling, SOCS3, and pro-IL-1β expression is inhibited by gamma secretase inhibitor DAPT **(A)** RAW cells (8x10^5^) were treated with various concentrations of DAPT (10, 25, and 50 µm in DMSO) overnight. The following day, cells were infected with DSV (MOI 20) for 7 hours. Cells were lysed and protein lysate was prepared. Fifty μg of protein lysate was separated on SDS-PAGE and analyzed for Notch1, NICD, SOCS3, IL-1β, and p21 by Western blotting. Actin was used as a loading control. **(B–F)** Quantification of Western blots. Blots were quantified with ImageJ by analyzing the ratio of protein of interest/Actin. Data represents Mean ± SEM from at least three independent experiments. One-way ANOVA was used to determine the statistical significance. Values were compared to control, with a post-hoc Dunnett’s test. **p < 0.01 and ***p < 0.001.

We pre-treated RAW cells with various doses of a gamma secretase inhibitor N-[N-(3,5-Difluorophenacetyl)-L-alanyl]-S-phenylglycine t-butyl ester (DAPT) followed by challenge with DSV (DSV+DAPT) (**Figure 3A**).Control cells were treated with equivalent volume of DMSO. The dose of DAPT was selected based on observations in RAW macrophages in other studies (Monsalve et al., 2009; Tsao et al., 2011). As expected, treatment of cells with DSV induced protein expression of Notch1 (DSV: 17.60 ± 3.688 *vs* control: 1.00±0.53, p<0.001) (**Figure 3A** left two panels & **3B**) and NICD (DSV: 4.65 ± 0.52 *vs* control: 1.00, p<0.001) (**Figures 3A, C**). We also examined the expression of p21, one of the target genes activated by NICD (Borggrefe and Oswald, 2009). DSV significantly induced p21 expression in RAW cells (DSV: 3.40 ± 0.33 *vs* control: 1.00±0.10, p<0.001) (**Figure 3D**). Similarly, we observed a significant increase in SOCS3 (DSV: 18.56 ± 6.14 *vs* control: 1.00±0.14p<0.01) (**Figures 3A, E**) and in pro-IL-1β expression by DSV (DSV: 83.3 ± 29.5116.95 *vs* control:1.00±0.53, p<0.01) (**Figure 3F**). Expression of Notch1, NICD, pro-IL1β, and p21 induced by DSV was significantly inhibited by DAPT at concentrations as low as 10µm (p values<0.05 for all proteins tested *vs*. DSV) (**Figures 3B–E**) including pro-IL-1β which was inhibited by DAPT at all concentrations tested (p value <0.05 *vs*. DSV alone) (**Figure 3F**) suggesting that DSV induces pro-IL-1β expression in a Notch signaling–dependent manner. While SOCS3 was inhibited a lower DAPT doses, statistical significance was observed at higher dose of DAPT 50μm when compared to DSV, p<0.05).

**Figure 4:**
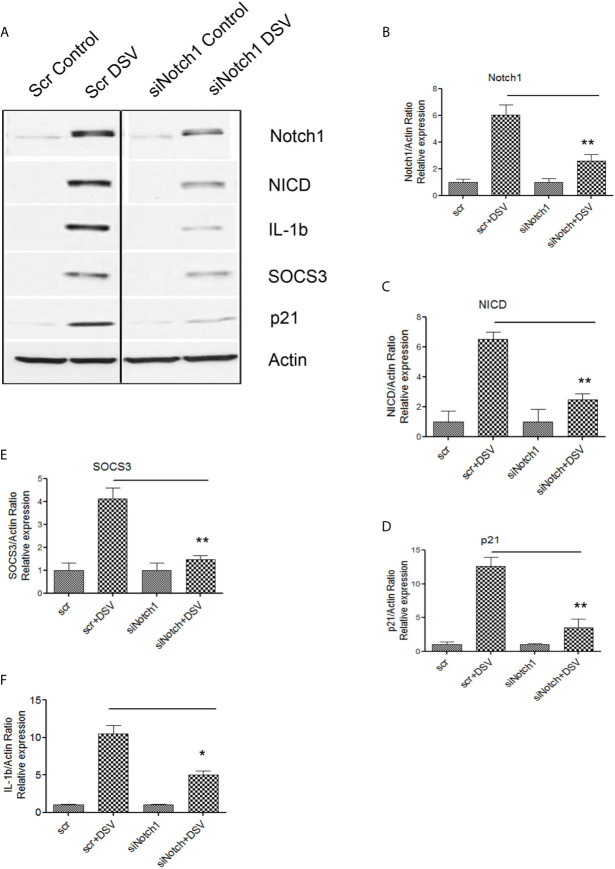
DSV-induced Notch signaling, SOCS3, and IL-1β expression is inhibited by Notch1siRNA **(A)** RAW cells were transfected with either scrambled (Scr) or siRNA against Notch1 (siNotch1) by electroporation. After incubation for 24 hours, cells were removed and re-plated at a density of 8x10^5^ and further incubated for 24 hours. Following day (total of 48 hrs transfection), cells were infected with DSV (MOI 20) for 7 hours. Cells were lysed and protein lysate was prepared. Fifty μg of protein lysate was separated on SDS-PAGE and analyzed for Notch1, NICD, SOCS3, IL-1β, and p21 by Western blotting. Actin was used as a loading control. **(B–F)** Quantification of Western blots. Blots were quantified with ImageJ by analyzing the ratio of protein of interest/Actin. Data represents Mean ± SEM from at least three independent experiments. One-way ANOVA was used to determine the statistical significance. Values were compared to Scr+ DSV, with a post-hoc Dunnett’s test. *p < 0.05, **p < 0.01.

We further confirmed these findings by using siRNA against Notch1. Cells were transfected with either scrambled control siRNA (Scr) or with siRNA against Notch1 (siNotch1) (**Figure 4A**). DSV induced expression of Notch1 and related proteins in cells transfected with Scr siRNA (Scr +DSV). In contrast, in cells knocked down for Notch1, the ability of DSV to activate Notch1-related proteins was significantly reduced (p values of siNotch1+DSV treatment groups<0.05 when compared to Scr +DSV treatment) (**Figures 4B–E**). Induction of SOCS3 by DSV was also reduced in siNotch1 transfected cells (p<0.05 compared to Scr+DSV) (**Figure 4E**). Expression of pro-IL-1β induction by DSV was also reduced in cells knocked down for Notch1 (Scr+ DSV: 10.49 ± 1.13 *vs* siNotch1+DSV: 5.01 ± 0.52, p<0.05) (**Figure 4F**). Taken together, these results suggest that DSV induces Notch1 pathway-related proteins, proinflammatory cytokine pro-IL-1β and SOCS3 cytokine regulator in macrophages in a Notch-signaling dependent manner.

We also confirmed these findings in mouse primary bone marrow-derived macrophages (BMMs) (**Figure S2**). BMMs were isolated and pre-treated with DMSO control or DAPT (10µm) followed by infection with DSV. Similar to RAW cells, DSV induced expression of Notch1, NICD, p21, SOCS3 and pro-IL-1β in BMMs when compared to untreated control cells. However, pre-treatment of cells with DAPT inhibited DSV-induced expression of these proteins, confirming our findings in RAW cells.

### DSV-Induced Notch1 Activation Occurs in TLR4-Independent Manner

**Figure 5:**
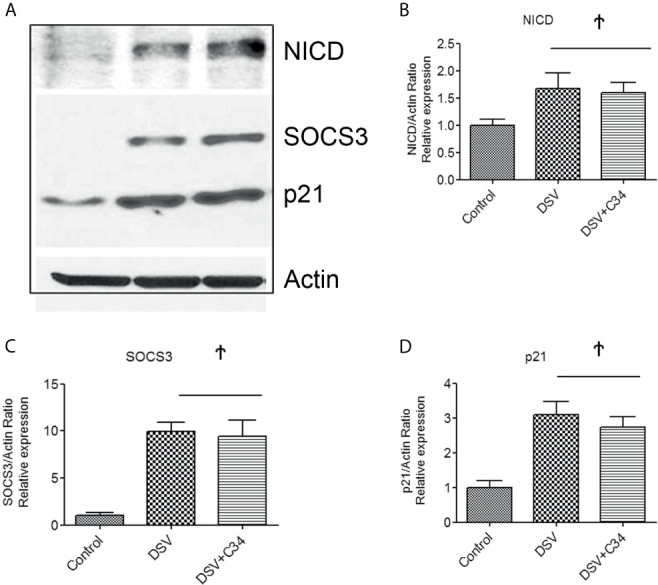
DSV-Induced Notch Activation occurs independent of TLR4 activation **(A)** RAW cells (8x10^5^) were first treated with 15µm C34, A TLR4 inhibitor for 30 mins prior to infection with DSV. Cells were harvested and protein lysate was prepared. Fifty μg of protein lysate was separated on SDS-PAGE and analyzed for NICD, SOCS3, and p21 expression by Western blotting. Actin was used as a loading control. **(B–D)** Quantification of Western blots. Blots were quantified with ImageJ by analyzing the ratio of protein of interest/Actin. Data represents Mean ± SEM from at least three independent experiments. Values were normalized to control. Ϯp > 0.05.

As Notch pathway has been shown to act cooperatively with TLR4 signaling (Hildebrand et al., 2018), we investigated whether Notch1 signaling induced by DSV required a functional TLR4 signaling pathway. TLR4 -IN -C34(C34) is a potent inhibitor of TLR4 signaling (Singh et al., 2020). We pre-treated RAW cells with TLR4-IN-C34 prior to infection with DSV. Compared to control cells, DSV induced a significant increase in NICD expression (DSV: 1.51 ± 0.03 *vs* control: 1.00±0.10, p<0.05). Pre-treatment of cells with C34 caused no inhibition of DSV-induced NICD expression (*vs* DSV+C34: 1.53± 0.18, p>0.05 compared to DSV) (**Figures 5A, B**). Similarly, pre-treatment with C34 had no inhibitory effects on DSV-induced SOC3 expression (DSV: 9.93± 0.95 *vs* DSV+C34: 9.4± 1.76, p>0.05) (**Figures 5A, C**) or p21 expression (DSV: 3.09± 0.37 *vs* DSV+C34: 2.73± 0.30, p>0.05) (**Figures 5A, D**). These findings demonstrated that DSV-activated Notch signaling occurred in a TLR4-independent manner.

### Paracrine Activation of Notch in Recipient Cells by Soluble Factors in Culture Supernatant of DSV-Treated RAW Cells

**Figure 6:**
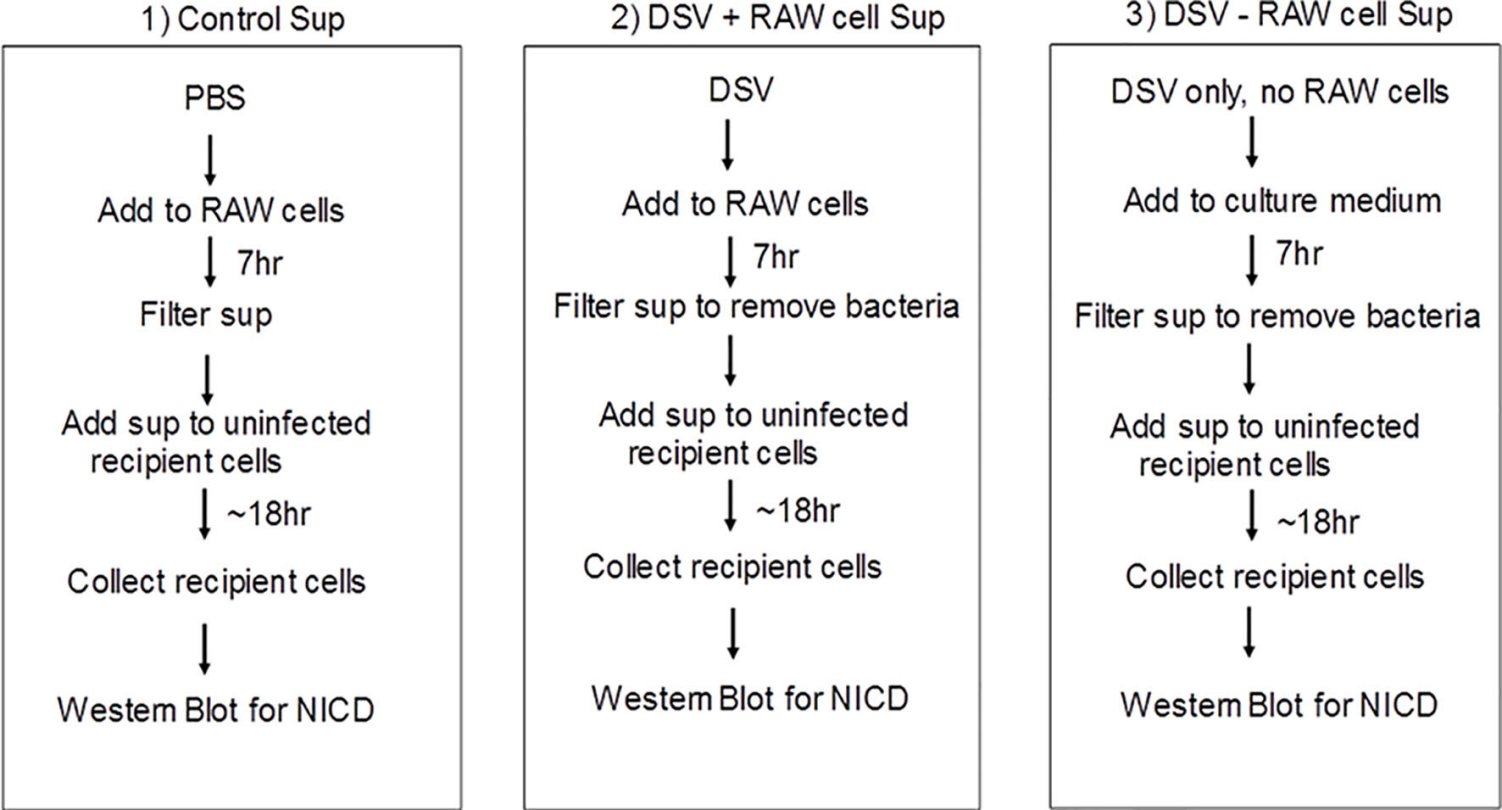
Schematic representation of the methodology for testing paracrine effect of soluble factors in DSV-infected cells on uninfected recipient cells.

**Figure 7:**
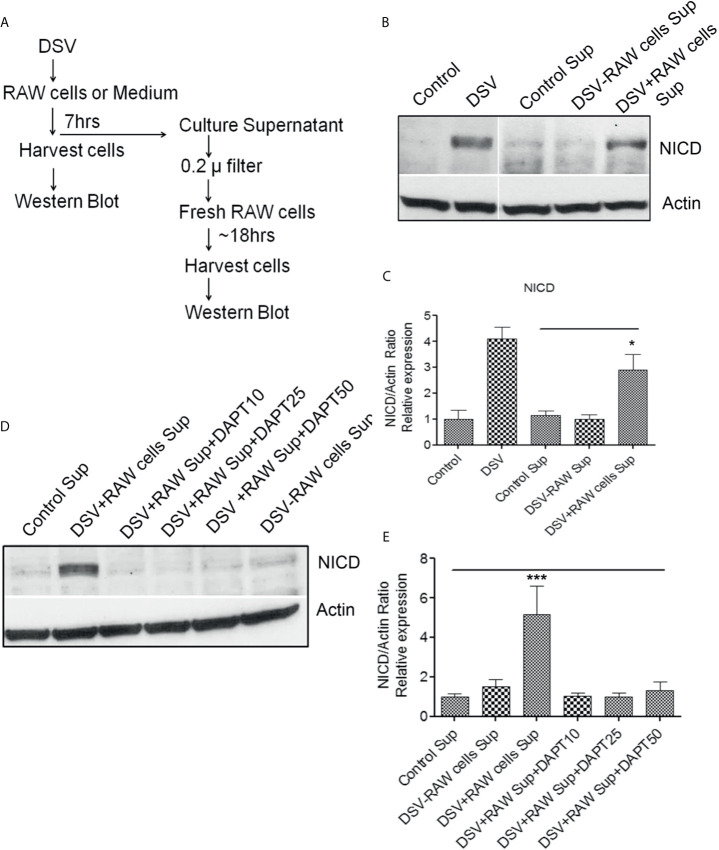
Paracrine activation of Notch in recipient cells by culture supernatant of DSV-treated RAW cells **(A)** Flow chart of the experimental strategy to examine the paracrine effects of DSV-Notch signaling on uninfected target cells. See **Figures 6**. **(B)** 8x10^5^ cells were plated the day before infection. Cells were infected with DSV (MOI 20) for 7 hours. Culture supernatants were collected and the infected cells were harvested and lysed. Control Sup, DSV-RAW cell sup, or DSV+RAW cell sup was passed through a 0.2 µm filter and added to a fresh plate of uninfected recipient cells overnight. The following day, the recipient cells were harvested and lysed. Fifty μg of protein lysate was separated on SDS-PAGE and analyzed for NICD expression by Western blotting. Actin was used as a loading control. **(C)** Quantification of Western blots. Blots were quantified with ImageJ by analyzing the ratio of protein of interest/Actin. Data represents Mean ± SEM from at least three independent experiments. One-way ANOVA was used to determine the statistical significance. Values were compared to control sup, with a post-hoc Dunnett’s test. *p < 0.05. **(D)** Same as in **(A)** but in this experiment, recipient cells were pre-treated with various concentrations of DAPT overnight. Medium was removed and replaced with either control sup, DSV-RAW cell sup or with DSV+RAW cell sup for overnight incubation. Fresh DAPT was again added to the cells along with the culture supernatants. **(E)** Quantification of Western blots. Blots were quantified with ImageJ by analyzing the ratio of protein of interest/Actin. Data represents Mean ± SEM from at least three independent experiments. One-way ANOVA was used to determine the statistical significance. Values were compared to control sup, with a post-hoc Dunnett’s test. ***p < 0.001.

Next, we examined whether Notch signaling initiated in DSV-infected macrophages could be transmitted to uninfected cells (Fresh RAW cells) *via* a secreted molecule(s) in the cell culture supernatant (Sup) (**Figures 6** and **7A**). We treated RAW cells with PBS or DSV for 7 hrs. (**Figure 6**; 1: Control Sup and 2: DSV+RAW cell Sup, respectively). In a separate plate, DSV was added for 7hrs to the RAW cell culture medium in a 6-well plate in the absence of RAW cells (**Figure 6**; 3: DSV-RAW cell Sup). After 7hrs, culture sups from the three treatments were collected. Infected cells were also harvested to confirm Notch activation by DSV (**Figure 7B**, left two lanes). All sups were filtered through a 0.2 µm filter before adding to the uninfected recipient cells. The filtered sup from PBS-treated cells (Control sup); 2: DSV-infected cells (DSV+RAW cells sup); 3: DSV only, no RAW cells (DSV-RAW cell sup) was added overnight to uninfected (fresh RAW) recipient cells. Recipient cells were then analyzed for NICD expression, as a marker of Notch activation. As expected, DSV infected cells (DSV) had an increased NICD expression when compared to untreated cells (Control) (**Figure 7B**, left two panels). When the effects of different sups on recipient cells were compared, we found that DSV+RAW cells sup elicited a significant NICD production in the recipient cells when compared to cells treated with control sup (DSV +RAW sup: 2.89 ± 0.60 *vs* Control Sup:1.00±0.34, p<0.05) (**Figures 7B, C**). In contrast, we did not observe induction of NICD in recipient cells treated with DSV-RAW cell sup (0.98 ± 0.18) when compared to control sup (1.00, p >0.05), suggesting that the source of NICD induction in recipient cells was a factor(s) secreted from DSV-infected RAW cells rather than a secreted product from DSV in RAW culture medium. Next, we pre-treated the recipient cells with various concentrations of DAPT overnight to inhibit Notch machinery in the recipient cells before adding the sups (**Figures 7D, E**). As expected, DSV+RAW cell sup (5.15 ± 1.43 *vs* control sup: 1.00±0.15, p<0.001), but not DSV-RAW cell sup (1.49 ± 0.36 *vs* control sup: 1.00±0.15, p>0.05), induced NICD expression in recipient cells when compared to the cells treated with control sup. However, DSV+RAW cell sup failed to elicit NICD expression in recipient cells that were pre-treated with DAPT (p>0.05 compared to control sup) at all concentrations of DAPT tested. These results suggest that paracrine activation of NICD in recipient cells by a secreted product in DSV+RAW cells sup was dependent on classical cleavage of Notch receptor in the recipient cells to achieve Notch activation in the those cells.

### DSV Induces NICD Expression in Epithelial Cell Lines and in Small Intestine

**Figure 8:**
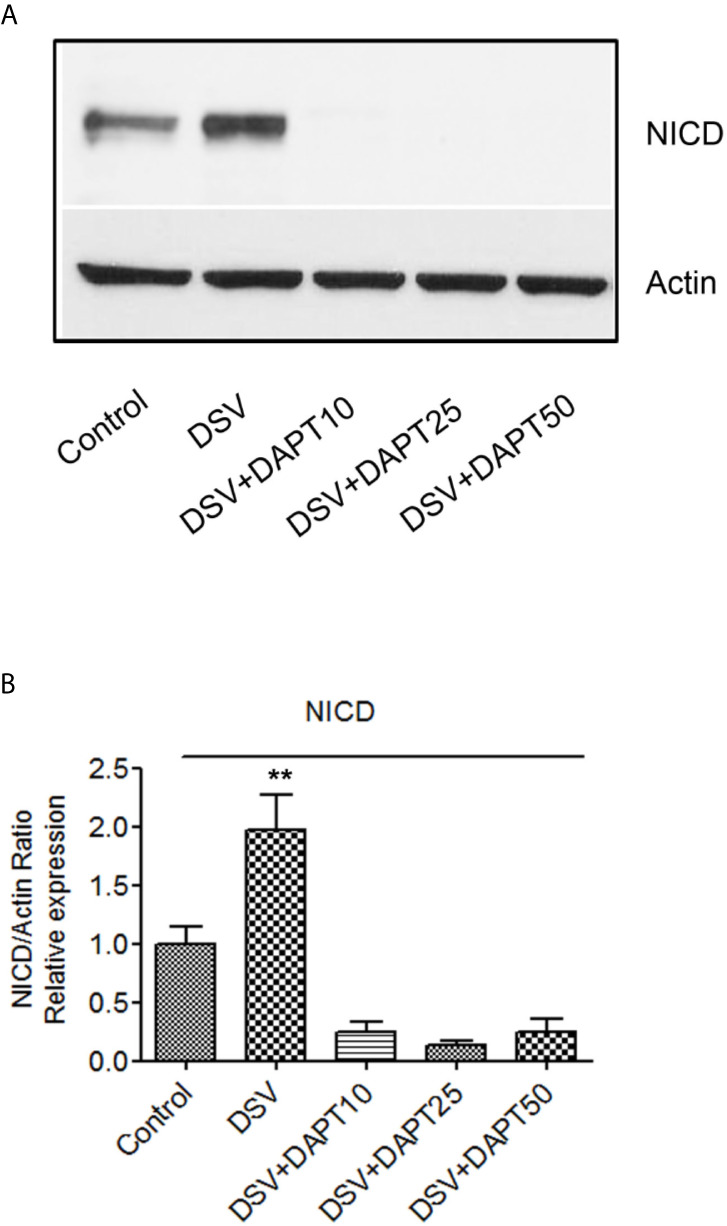
DSV induces Notch activation in epithelial cells **(A)** HCT116 cells (8x10^5^) were infected with DSV at MOI 20 for 7 hours. Cells were lysed and protein lysate was prepared. Fifty μg of protein lysate was separated on SDS-PAGE and analyzed for NICD and Actin by Western blotting. **(B)** Quantification of Western blots. Blots were quantified with ImageJ by analyzing the ratio of NICD/Actin. Data represents Mean ± SEM from at least three independent experiments. Values were normalized to control. **p < 0.01.

**Figure 9:**
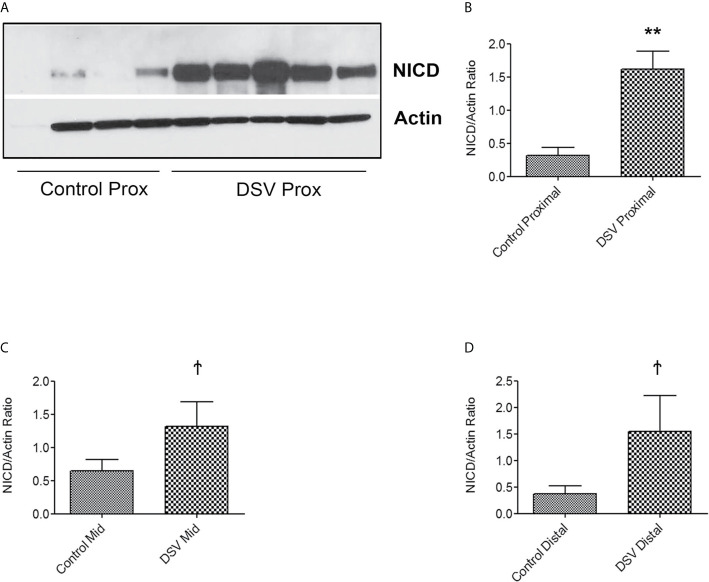
DSV induces NICD activation in the mouse small intestine Mice were orally gavaged with either PBS or DSV (10^9^). One hour later, mice were euthanized and 1/3^rd^ parts of small intestinal tissue corresponding to Prox (duodenum), Mid (Jejunum), and Distal (ileum) regions were collected in Trizol. Protein samples (10µg) were prepared and analyzed by Western blotting for NICD expression. Actin was used as a loading control. **(A)** NICD expression in protein samples from 4 control and 5 DSV-treated tissues **(B–D)** Quantification of Western blots. Blots were quantified with ImageJ by analyzing the ratio of NICD/Actin. Data represents Mean ± SEM. **p < 0.01, Ϯp > 0.05.

We also tested whether DSV induced NICD expression in epithelial cells. For this, human colonic epithelial cell line HCT116 was infected with DSV (MOI 20) for 7 hours. Cells were lysed and probed for NICD expression (**Figures 8A, B**). Similar to RAW macrophages, DSV-treated HCT116 cells showed a higher expression of NICD (1.96 ± 0.30 when compared to control untreated cells (1.00±0.14, p<0.01) and this effect was inhibited in cells pretreated with all concentrations of DAPT (p<0.001, compared to DSV). Activation of NICD in response to DSV was also assessed in whole animals. Mice were orally gavaged with either PBS (Control) or with 10^9^ DSV (N=6/group) for 1hr following which the animals were euthanized and small intestinal tissues collected as 1/3^rd^ proximal, mid and distal regions corresponding to duodenum, jejunum, and ileum fractions, respectively. One hour time point was selected based on our previous study with DSV (Ritz et al., 2017). Tissue samples were processed for Western blotting to detect NICD activation (**Figure 9**). We found that in the proximal small intestine, there was a significant increase in NICD expression in DSV-infected mice when compared to control mice (DSV Proximal: 1.63 ± 0.26 *vs* Control Proximal: 0.27 ± 0.09, p<0.01) (**Figures 9A, B**). While there was an increase in the mid and distal small intestinal sections in DSV- treated mice compared to control mice, the effects were statistically insignificant (**Figures 9C, D**). We also tested the expression of IL-1β in tissue samples by ELISA (**Figure S3**). We found that there was a trend for increase in IL-1β expression in DSV-treated proximal tissues, however, the difference was statistically insignificant. This may be attributed to the fact that the DSV gavage was allowed for one hour and the inflammatory effects were only beginning to appear within this time frame.

## Discussion

In this study, we report activation of Notch signaling pathway by sulfate reducing bacteria (SRB) as a novel mechanism by which these bacteria communicate with host cells. We showed that *Desulfovibrio vulgaris* (DSV), a member of SRB family, induced Notch1 signaling in RAW 264.7 macrophages, in mouse primary BMMs, in HCT116 epithelial, and in mouse small intestine. DSV also induced the expression of proinflammatory cytokine pro-IL-1β and a cytokine signaling regulator SOCS3, which was inhibited by DAPT or by Notch1 siRNA. Additionally, secretory factors in the culture supernatants of DSV-treated macrophages induced NICD expression in uninfected recipient cells, an action that depended on functional Notch machinery in the recipient cells.

SRB, specifically *Desulfovibrio* species, have been reported to increase in number in conditions such as ulcerative colitis (UC), periodontitis, pouchitis, metabolic syndrome, and obesity (Singh and Lin, 2015) and in antibiotic-treated mouse model (Singh et al., 2017). However, the underlying mechanisms of the association between SRB and these diseases and the effects of SRB on host cellular mechanisms remain largely unexplored. A few studies have demonstrated the effect of SRB on host cells to understand the role of SRB in pathophysiology of diseases. In a recent study, it was demonstrated that colonization of germ free mice with *Desulfovibrio indonesiensis* or with human SRB consortium caused alterations such as increased number of Th17 and Treg cells (Figliuolo et al., 2017). Additionally, LPS isolated from *D. desulfuricans* was found to cause an increase in pro-inflammatory cytokine production (Weglarz et al., 2003). SRB isolated from UC patients were found to induce apoptosis in epithelial cells *in vitro* (Coutinho et al., 2017). These findings suggest that SRB or their products may be the contributing factors in adverse outcomes such as those observed in inflammatory conditions associated with a SRB bloom.

Notch pathway is a highly conserved cell-cell communication pathway involved in tissue development and differentiation. In the intestine, Notch determines the secretory fate of intestinal cells such as Paneth cells and goblet cells (Noah and Shroyer, 2013) and inhibition of Notch signaling by gamma secretase inhibitor induces production of goblet and enteroendocrine cells (Wong et al., 2004). In addition, gut microbes have been reported to determine the fate of secretory cells by modulating Notch signaling (Troll et al., 2018). Dysregulation of Notch pathway has been linked to diseases such as IBD, cancer, and autoimmune diseases (Demarest et al., 2008; Okamoto et al., 2009). Overexpression of Notch pathway genes has been observed in DSS-colitis mouse models (Vooijs et al., 2011), and active Notch signaling is associated with a variety of inflammatory conditions such as rheumatoid arthritis (Park et al., 2015), atherosclerosis (Aoyama et al., 2009), and infections (Narayana and Balaji, 2008; Ito et al., 2011). Blockade of Notch signaling was found to prevent inflammatory gene expression (Poulsen et al., 2018) and ameliorate arthritis in a mouse model (Jiao et al., 2014), and. These studies suggest that the Notch pathway is involved in the development of inflammatory conditions. Notch is also activated in macrophages in response to various pathogenic bacteria and microbial products such as *Mycobacteria bovis BCG* (Narayana and Balaji, 2008), tuberculin purified protein derivative (Palaga et al., 2013), *Ehrlichia chaffeensis*  (Lina et al., 2016), and LPS (Bai et al., 2018). Notch signaling regulates immune responses in macrophages including production of pro-inflammatory cytokines (Levi, 2017). Specifically, overexpression of NICD in RAW cells increased expression of TNFα (Monsalve et al., 2009), and inhibition of Notch signaling in primary bone marrow-derived macrophages decreased LPS and IFNγ induced expression of TNFα and iNOS (Palaga et al., 2008). Our observation that DSV induces pro-IL-1β and SOCS3 expression in a Notch-dependent pathway in macrophages is consistent with these studies.

How dysregulated Notch pathway in the epithelial cells as well as myeloid cells contributes towards colits, remains to be characterized. It was shown that in DSS and TNBS- induced model of colitis, Notch ligands and receptors were upregulated in colonic epithelial cells in a TLR5 -dependent manner (Aziz et al., 2013). Whether or not DSV induces Notch activation *via* TLR5 remains to be determined. Role of Notch signaling in macrophages in context of inflammation has also been reported in many studies (Fung et al., 2007; Bai et al., 2018). It was shown that Notch myeloid-specific knockdown of Notch inhibited pro-inflammatory cytokines which ameliorated organ dysfunction associated with sepsis (Bai et al., 2018). A positive correlation was observed between M1/M2 ratio and Notch signaling in mucosa of CD patients (Ortiz-Masiá et al., 2016). It will be important to understand how Notch activation in cell types used in this study would participate in inflammatory conditions, in the setting of DSV bloom.

Previous studies showed that LPS induced the expression of Notch ligands and receptors *via* TLR4 activation (Hildebrand et al., 2018). Since SRBs could be a potential source of LPS, we tested whether DSV-induced Notch signaling depended on LPS working through TLR4 pathway. In this study, inhibiting TLR4 by TLR4–IN-C34 inhibitor did not block DSV-induced Notch signaling suggesting that this response to this SRB was independent of TLR4 activation. SRB also generate hydrogen sulfide (H_2_S) as their metabolic byproduct. The H_2_S generating molecule sodium hydrosulfide (NaHS) has been shown to activate Notch pathway by increasing Jag1 and Hes1 in the hippocampus in a vascular dementia rat model (Liu et al., 2016). However, it is unlikely that DSV continuously generates H_2_S in the mammalian culture medium in the absence of strict anaerobic conditions and an appropriate substrate. Future studies are needed to understand the mechanism of how DSV induces Notch signaling in host cells.

In addition to live DSV, we found that heat killed DSV also induce NICD, p21, and pro-IL-1b expression in RAW cells comparable to live DSV. These results suggest that a structural component rather the metabolically active DSV is responsible for activating Notch pathway in macrophages. Future studies will focus at identifying the components of DSV that are responsible for activating Notch signaling in host macrophages.

Notch ligands and receptors may be differentially expressed in a cell-specific manner, and may regulate Notch signaling in this context (Radtke et al., 2010). Notch ligands and receptors are also expressed in immune cells such as macrophages (Jonsson et al., 2001). DSV induced gene expression of DLL1, Jagged1 (Jag1), and Notch1 but inhibited DLL3, DLL4, Jagged2 (Jag2), and Notch 4 suggesting that DSV differentially regulates the expression of Notch ligands and receptors. Notch 4 appears to have an opposite effect to Notch1 (James et al., 2014), which explains DSV activation of Notch1 while inhibiting Notch 4. An increase in DLL1 by DSV is supported by the findings that DLL1 is upregulated in monocytes during bacterial infection (Hildebrand et al., 2018). Jag1 is known to inhibit Notch 4 signaling in endothelial cells (Benedito et al., 2009). The same study also found that pro-inflammatory TNFα induced Jag1 and inhibited DLL4 transcription. DLL3 was reported previously to inhibit Notch signaling (Ladi et al., 2005). Thus, receptor ligand interactions may be dependent on cell and experimental context. These data support differential expression of Notch ligands and receptors in DSV-regulated Notch signaling.

We also observed an increase in the expression of SOCS3 protein in response to DSV. SOCS3 has been shown to depend on Notch activation in cells treated with *M. bovis* BCG. Overexpression of NICD was reported to enhance SOCS3 induction by *M. bovis* BCG (Narayana and Balaji, 2008). These reports support our data showing induction of SOCS3 expression by DSV in a Notch-dependent manner. Induction of suppressor of cytokine signaling (SOCS3), an intracellular protein that inhibits immune response, has been reported to be triggered by LPS from gram negative bacteria to reduce TLR4-dependent inflammatory response (Berlato et al., 2002; Duncan et al., 2017). A similar role is possible for SOCS3 triggered by DSV.

We also found that supernatant from DSV-infected RAW cells filtered to remove DSV elicited Notch activation in uninfected recipient macrophages. This suggests a mechanism by which DSV bloom in the gut may be relayed from the originating cell to neighboring cells *via* activation of Notch signaling. Only the culture supernatant of DSV-treated cells (DSV+RAW cell sup) but not DSV-RAW cell sup enabled NICD activation in recipient cells. This suggests that NICD activating factor was most likely derived from RAW cells responding to DSV and not secreted by the bacteria directly. The soluble factor responsible for activating Notch signaling in recipient cells remains to be determined. Notch ligand DLL1 has been found in the plasma of sepsis patients and the soluble form of DLL1 was observed in cell supernatants of human monocytes (Hildebrand et al., 2019). It is therefore conceivable that soluble DLL1 may be one of the factors responsible for the effect observed in our study.

We also found that DSV induced significant expression of NICD in the proximal region of the small intestine but not significant in mid and distal region. This could be attributed to the time point selected for gavage. One hour of gavage was selected based on our previous studies that showed that gavage with DSV close to 1 hr induced slowing of intestinal transit (Ritz et al., 2017) as well as changes in cellular gene and protein expression in the small intestine (Singh et al., 2017). In vivo, we measured NICD activation in whole tissue samples which included many different cell types other than macrophages. It is likely that overall physiological response of total intestinal cells is much faster to an insult. Additionally, presence of possible host and other microbial response factors in the intestine may signal the host about the presence of this bacteria. Thus, *in vivo* cellular response to DSV is far more complicated and is likely to differ in the kinetics of Notch induction to DSV in comparison to the pure cell culture where we observed a significant induction of NICD at a later time point.

Main limitation of our study is that we did not examine in details the inflammatory response to DSV and whether it depends on Notch signaling. Future studies will focus on addressing these issues to better understand the relationship between Notch activation and inflammation in response to DSV.

In conclusion, our study reveals a novel mechanism by which sulfate reducing bacteria (SRB) interact with host cells by activating Notch signaling pathway that further controls the protein expression of SOCS3 and pro-IL-1β, molecules involved in inflammatory responses. Controlling Notch activation in response to SRB may be helpful in controlling adverse outcomes associated with SRB bloom.

## Data Availability Statement

The original contributions presented in the study are included in the article/**Supplementary Material**. Further inquiries can be directed to the corresponding author.

## Ethics Statement

The animal study was reviewed and approved by Institutional Animal Care and Use Committee at the New Mexico VA Health Care System.

## Author Contributions

SS conceptualized the study, designed, and performed experiments, analyzed the data, and wrote the manuscript. CC, AC-P and MV helped with experimental design and performed the experiments. HL conceptualized and designed the study and edited the manuscript. All authors contributed to the article and approved the submitted version.

## Funding

This study was supported by the Winkler Bacterial Overgrowth Research Fund, grant number BRINM 217.

## Conflict of Interest

The authors declare that the research was conducted in the absence of any commercial or financial relationships that could be construed as a potential conflict of interest.
