# Supplementary material for: Notch Signaling Pathway Is Activated by Sulfate Reducing Bacteria
Source: Front Cell Infect Microbiol. 2021 Jul 15;11:695299. doi: 10.3389/fcimb.2021.695299 (PMC8319767; doi:10.3389/fcimb.2021.695299)
Supplement: Supplementary file 1 [file DataSheet_1.docx]

**Figure. S1 Heat killed DSV induces Notch pathway and pro-IL-1β expression in RAW cells.** DSV was heat killed by autoclaving the bacterial culture. Bacteria were counted before heat killing and volume equivalent to MOI20 was added to RAW cells plated in 6-well plates for 7 hours. Cells were also infected with live DSV. Cells were lysed and protein lysate was prepared. Fifty μg of protein lysate was separated on SDS-PAGE and analyzed for NICD, pro-IL-1β, and p21 by Western blotting. Actin was used as a loading control. Blots were quantified using imageJ. Values were calculated by normalizing against untreated controls. Data represents mean±SEM from three independent experiments. *p<0.05, **p<0.01.

**Figure. S2 DSV induces Notch pathway and pro-IL-1β expression in primary Bone Marrow-Derived macrophages** Bone Marrow-derived macrophages were isolated and pooled from 3 mice. Cells were counted and plated in 6-well plates. Cells were infected with DSV (MOI 20) for 7 hours. Cells were lysed and protein lysate was prepared. 15 μg of protein lysate was separated on SDS-PAGE and analyzed for Notch1, NICD, SOCS3, pro-IL-1β, and p21 expression by Western blotting. Actin was used as a loading control.


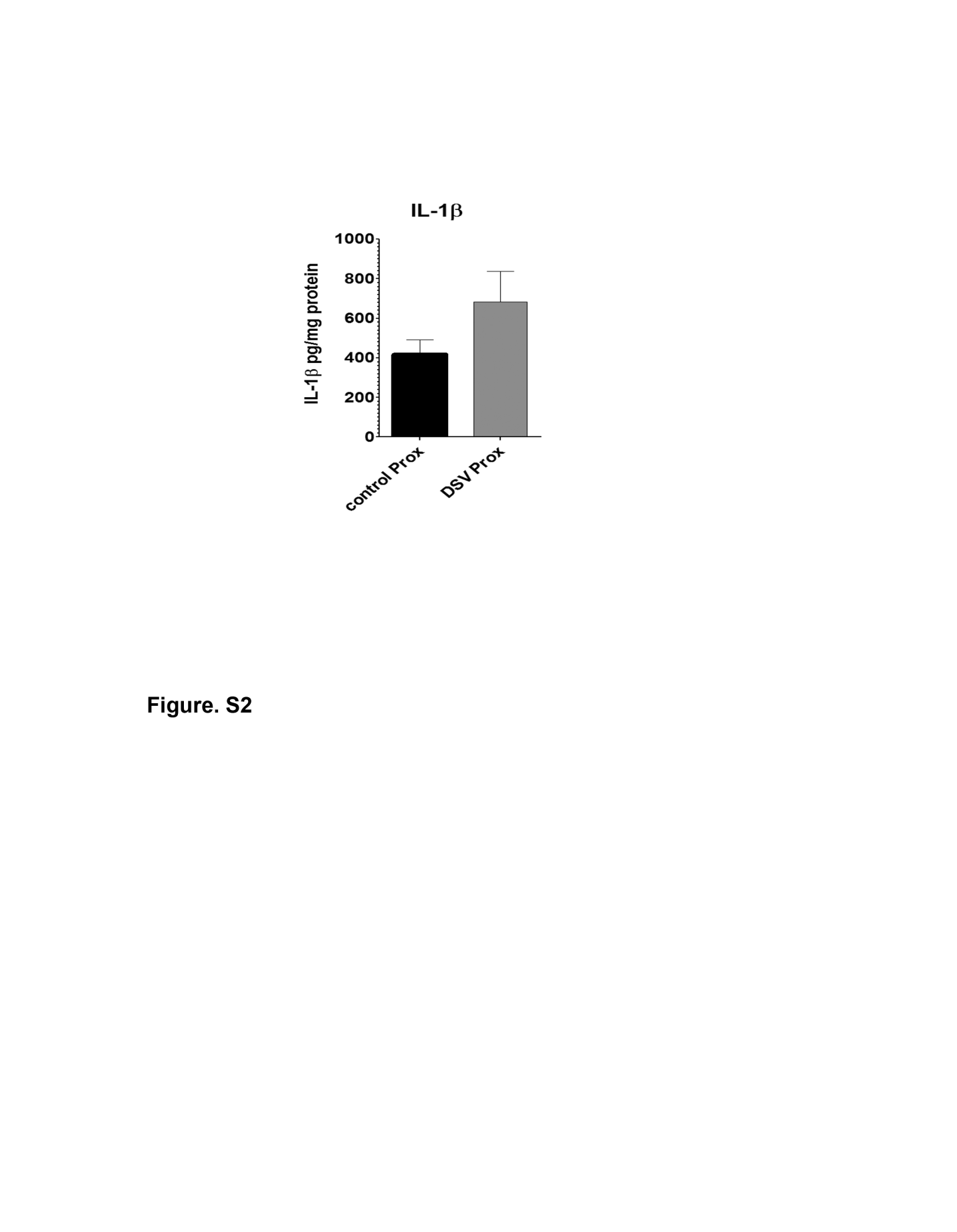


**Figure.S3 Increased IL-1β production in DSV- treated Proximal tissues.**  IL-1β was analyzed by ELISA in protein samples isolated from control or DSV- treated proximal tissues using manufacturers’ instructions. Concentration of IL-1β was measured using standards for IL-1β provided in the ELISA kit. Values were plotted as amount of IL-1β in pg/mg of protein sample (Mean±SEM, N=6/group).
